# One-minute through test to distinguish lower respiratory infection by analysis of sputum; exploring the mechanisms

**DOI:** 10.1186/s13104-018-3771-1

**Published:** 2018-09-12

**Authors:** Amir Ramezani, Mahin Alipouratigh, Lars Eng, Maria V. Turkina, Johanna Lönn, Annette Theodorsson, Fariba Nayeri

**Affiliations:** 1 Division of Neuro and Inflammation Science, Department of Clinical and Experimental Medicine, Linköping University, Linköping, Sweden; 2 Department of Microbiology, Regional Council of Östergötland in the Southeast of Sweden, Linköping, Sweden; 3 The Institute for Protein Environmental Affinity Surveys (PEAS Institute), Linköping, Sweden; 4 Department of Clinical and Experimental Medicine, Linköping University, Linköping, Sweden; 5 Department of Oral Biology, Faculty of Odontology, Malmö University, Malmö, Sweden

**Keywords:** Respiratory infection, Diagnosis, Neutrophil extracellular traps, Cell-free DNA, Dische’s test

## Abstract

**Objective:**

Cough and fever are the initial symptoms of lower respiratory infection. Severe cases might be fatal. Therefore, particularly in the non-equipped centers, the lack of diagnostic methods to identify the severe cases has resulted in overconsumption of antibiotics. On the basis of the knowledge about non-specific immune response at the site of injury, we developed a colorimetric dip-test that shows abrupt, sensitive and quite specific color change upon contact with sputum in the cases of lower respiratory infection. We further explored the mechanism of the test.

**Results:**

We detected deoxyribonucleic acid (DNA) and hepatocyte growth factor in the sputum of patients that suffered from respiratory infection (n = 18). The results differed significantly (*P *< 0.0001) from age-matched patients (n = 18) with other respiratory disorders and highly correlated with the index-test results (Spearman Rank test = 0.84). DNA with a concentration more than 0.03 mg/ml induced a visible and stable color change on index-test within 1 min. The test recognized all of the cases with respiratory infection and the specificity was 72%. With a high negative predictive value. The index test detects, inter alia, cell-free DNA in sputum and might safely rule-out respiratory infection in 2/3 of cases that present symptoms of acute respiratory infection.

**Electronic supplementary material:**

The online version of this article (10.1186/s13104-018-3771-1) contains supplementary material, which is available to authorized users.

## Introduction

Deadly lung damage can occur during a lower respiratory infection. Timely diagnosis of diseases is crucial for decreasing the mortality rate [1]. The initial reaction of the innate immunity system at the site of injury—can be used for diagnostic purpose. As discovered recently among its task is to activate neutrophils to extrude their chromatin out of the cell in order to trap and kill bacteria [2]. These “neutrophil extracellular traps” (NETs) correlate strongly to the severity of respiratory infection [2]. NETs is composed of proteins and nucleic acid [2]. Also coinciding with pneumonia are elevated levels of hepatocyte growth factor (HGF) [3, 4] which exhibits high affinity to sulfated glycan as reported previously [5].

A mixture composed of sulfated glycan and toluidine blue can be employed to detect the cell-free nucleic acid in extravascular body fluids. This report presents the results from assessment of the mechanism of this solution (index-test) to distinguish infection, from other respiratory disorders.

## Main text

### Materials and methods

#### Sputum samples

Healthy individuals do not cough up enough sputum. The clinical evaluation (submitted article) and the technology of the index-test was assessed by analysis of left-over sputum that were used for sputum cultures (between November 1 2015 and January 30 2016). The samples were collected from patients in sterile tubes (Sarstedt) and within 24 h delivered to the laboratory. Within 30 min after arrival the samples were mixed 1:1 with Sputolysin (CAS 578517, diluted 1:10 in MQ water; Calbiochem). The tubes were then vortexed for 20 s and cultured within 15 min. The leftover samples after culturing, were kept at 4 °C until they were collected and coded by the study nurse in the evening. The code was consisted of year-month-day of birth-the third number in 4-digit code in the personal identification number. The index test was performed within 72 h on coded samples by two members in the study group and the results were documented in Excel-file. The complementary diagnostic procedures on the patient were selected by the physician in charge on ward with no relation to the study group. From April to June 2016, a physician and the study nurse reviewed the journals. The clinical symptoms, the blood and sputum cultures and PCR, the X-rays, the antibiotic therapy, and the ultimate diagnosis code (ICD-10) were documented in Excel-files. Sputum samples (n = 49) from patients born 1961–2008 were aliquoted in small tubes, kept frozen − 135 °C and used for cytokine surveys. In 2018, available sputum samples (n = 37) were thawed and re-analyzed. One sample could not be identified in the Excel-fil and was excluded. We compared two groups composed of patients with diagnosed respiratory infection (n = 18, born 1961–2008 median age 1979) and patients with respiratory symptom in which infection in the respiratory tract was not verified (n = 18, born 1961–2008, median 1974) (Additional file 1: Table S1).

#### Methods

HGF was determined by ELISA (Quantikine; R&D systems). Detection of nucleic acids was performed by UV–vis spectrophotometry and staining agarose gels with 1:5000 CYBR green dye for both single and double-strand DNA after electrophoresis. Dische’s test [6] was used for detection and quantification of DNA. Analysis by index dip-test using the dextran sulfate-toluidine blue solution [7] immobilized on custom filters was used.

#### Statistics

The differences and correlation between HGF concentration, DNA amounts determined by Dische’s test and index test analysis were examined using Kruskal–Wallis and Mann–Whitney U test. Correlation between parameters was analyzed by Spearman’s Rank test (GraphPad software, La Jolla, CA, USA). The sensitivity, specificity, negative predictive value and positive predictive value were analyzed (Medcalc).

### Results

**Fig. 1:**
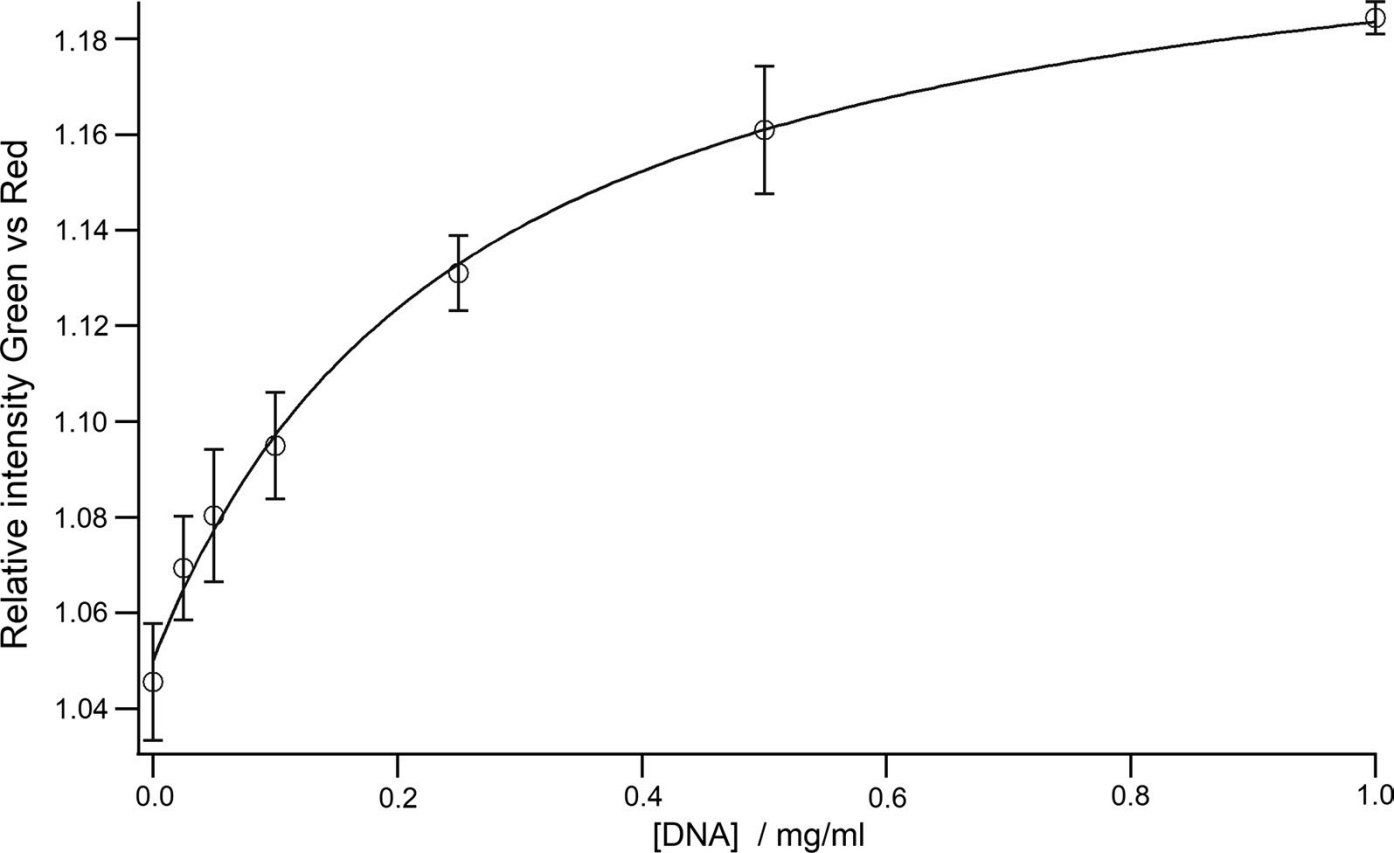
Standard curve for detection of known concentration of DNA by index dip-test and using digital in-house RGB reader

**Fig. 2:**
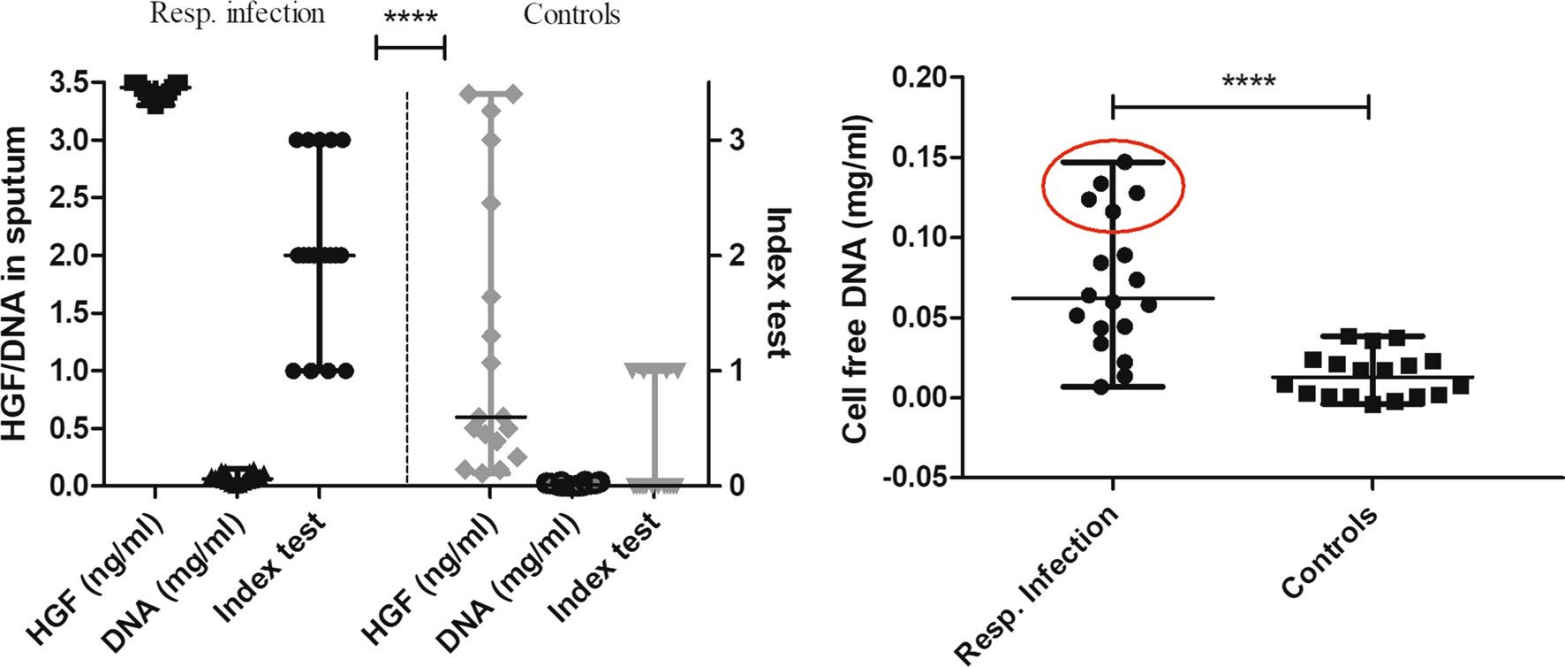
Left histogram shows the three indicators in sputum; HGF, cell-free DNA and index dip-test in the groups respiratory infection and controls. Right histogram shows the difference between groups in cell-free DNA concentration. The red circle separates the cases that had more severe symptom than the rest of patients. Values are expressed as median (min–max) and *P* ≤ 0.05 was considered as statistically significant. Significance is denoted as: *P < 0.05, **P < 0.01, ***P < 0.001 and ****P < 0.0001

UV-spectrometry, at 265 nm distinguished cases with respiratory infection (Additional file 2: Figure S1). Staining of agarose gels run with sputum samples from patients with respiratory infection before and after ultra-filtration in 30 kDa filters, showed presence of dsDNA in retentate (Additional file 3: Figure S2). DNA with a concentration more than 0.03 mg/ml induced a visible and stable color change on index dip-test within 1 min (Fig. 1). There were significant differences in HGF concentration, DNA concentration and index dip-test results between the patients with respiratory infection and patients with other respiratory disorders (P < 0.0001) (Fig. 2). The results from index dip-test correlated highly to sputum-DNA and -HGF concentrations (0.84). The index dip-test could identify a respiratory infection with 100% sensitivity and negative predictive value, and 72.2% specificity (95% CI 46.5–90.3%) (Table 1).

**Table 1:** Analysis of the index dip-test ability to distinguish respiratory infection shows 100% sensitivity (95% CI 81.4–100%), 72.22% specificity (95% CI 46.52–90.31%), 100% negative predictive value and 78.26% (95% CI 63.09–88.35%)

Index test	Present	n	Absent	n	Total
Respiratory infection					
Positive	True positive	a = 18	False positive	c = 5	a + c = 23
Negative	False negative	b = 0	True negative	d = 13	b + d = 13
Total		18		18	36

Diseases prevalence 50%

### Discussion

Since Brinkmann et al. in 2004 [8] discovered a previously unknown role of neutrophils during innate immunity, several researchers have studied NETs during infectious and autoimmune diseases [9]. Based on the knowledge about the high affinity of aniline dyes such as toluidine blue to DNA/nucleic acids, described by Paul Ehrlich 150 years ago, we have established a colorimetric method that detects and quantifies the cell-free DNA in sputum with high correlation to DNA concentration and high accuracy to distinguish lower respiratory infection from other non-serious disorders.

The reaction is visible within 1 min. Even though the results are optimally documented by using a color reading device, the reaction can also be interpreted by the naked eye. The test solution is stable at room temperature for several years and the sputum can be kept at room temperature due to the stability of DNA in the samples. The test solution is non-toxic, can be discarded as infective waste and its production costs are low.

### Conclusion

The presented diagnostic platform has the potential to improve the diagnosis of severe respiratory infections and facilitate the proper use of antibiotics in respiratory infections. There might be future indications in forensic medicine and archeology.

## Limitations

Studies on the early differences between bacterial respiratory infection and other respiratory disorders are of high value for development of new feasible, accurate and affordable platforms. We have presented a platform that should be assessed by more clinical studies in different settings in order to recognize the true value of such test in diagnosis, empiric antibiotic administration and monitoring of pneumonia. Besides, studies on activated neutrophils in fresh sputum might give further valuable information to understand the mechanism of reactions.

## Additional files

**Additional file 1: Table S1.** The detained information about included cases in the study both with respiratory infection and controls.

**Additional file 2: Figure S1.** UV-vis spectrophotometry of sputum samples from patients with respiratory infection (upper image) shows an increased absorbance at 265 nm (chromatin) compared to controls (lower image).

**Additional file 3: Figure S2.** Agaros gel electrophoresis of samples. The gel was dyed with 1:5000 CYBR green dye for both single and double-strand DNA. The second lane on left is the renentate from the pus from tooth abscess that was centrifuged 60,000 rpm in 60 min in ultra-filtration 30 kDa filters (index test highly positive), the fourth lane on left is 0.5 ml air-dried sputum sample reconstituted in 100 µl MQ (index test positive). The sixth lane on left is the renentate from the sputum sample that was centrifuged 60,000 rpm in 60 min in ultra-filtration 30 kDa filters (index test highly positive). The first lane on the right is the filtrate from same sputum sample (index test negative). The experiment was repeated six times.

## Abbreviations

DNA: deoxyribonucleic acid
NETs: neutrophil extracellular traps
HGF: hepatocyte growth factor
ELISA: enzyme-linked immunosorbent assay
UV–vis spectrophotometry: UV–visible spectrophotometry
