# Supplementary material for: One-minute through test to distinguish lower respiratory infection by analysis of sputum; exploring the mechanisms
Source: BMC Res Notes. 2018 Sep 12;11:664. doi: 10.1186/s13104-018-3771-1 (PMC6134600; doi:10.1186/s13104-018-3771-1)
Supplement: Supplementary file 1 — Additional file 1: Table S1. The detained information about included cases in the study both with respiratory infection and controls. [file 13104_2018_3771_MOESM1_ESM.docx]

| Microbiological assessment Sputum | Index test composed of three surfaces with different composition ratio | HGF ng/ml  sputum | DNA sputum  µg/ml | Diagnosis | Birth /sex |
| --- | --- | --- | --- | --- | --- |
| *Streptococcus pneumoniae,*  *Staphylococcus aureus* | 0 | 1.07 | 7 | Colonization of bacteria/no infection | 1981/male |
| *Mycoplasma pneumoniae* | **3** | **3.50** | **133** | **Pneumonia, intravenous antibiotics, several changes** | **1969/male** |
| Negative | 0 | 0.39 | 0 | Control post pneumonia | 1961/male |
| *Staphylococcus aureus* | 0 | 0.14 | 0 | Cystic fibrosis control after therapy | 2008/male |
| *Staphylococcus aureus* | **2** | **3.42** | **22** | **Cystic fibrosis, acute infection, oral antibiotics, aminoglycoside inhalation** | **2008/male** |
| Negative | 0 | 0.5 | 0 | Control post pneumonia | 1991/female |
| Negative | 0 | 0.11 | 0 | Control post pneumonia | 1978/male |
| *Moraxella catarrhalis* | **1** | **3.4** | **13** | **Bronchiectasis, giving birth, oral antibiotics** | **1977/female** |
| *Candida albicans* | 1 | 0.25 | 1 | Cystic fibrosis, control | 1997/female |
| Negative | 0 | 0.45 | 0 | Control post pneumonia | 1985/female |
| *Staphylococcus aureus, Pseudomonas aeruginosa*  *Haemophilus influenza*  *Candida albicans* | **2** | **3.5** | **63** | **Cystic fibrosis, exacerbation, intravenous antibiotics** | **1987/female** |
| Negative | 1 | 1.3 | 17 | halitosis | 1973/male |
| Negative | **1** | **3.3** | **33** | **Immunodeficiency, coughing and sore throat, oral antibiotics** | **1979/female** |
| *Haemophilus influenza* | **2** | **3.55** | **89** | **Bronchitis, oral antibiotics** | **1981/female** |
| Negative | 0 | 0.14 | 24 | Control post pneumonia | 1999/female |
| Negative | 0 | 0.6 | 20 | Control post pneumonia | 1961/male |
| *Moraxella Catarrhalis* | **3** | **3.45** | **147** | **Immunodeficiency, Mb Bruton, bronchitis, therapeutic failure, combining two antibiotics** | **1981/male** |
| Negative | 0 | 1.64 | 2 | Diabetes, SLE, renal insufficiency, control | 1984/male |
| *Pseudomonas aeruginosa, Staphylococcus aureus* | **2** | **3.6** | **84** | **Cystic Fibrosis, exacerbation**  **New cultures, no antibiotics** | **1977/female** |
| *Staphylococcus aureus, Exophilia art* | **2** | **3.5** | **73** | **Cystic Fibrosis, exacerbation, intravenous antibiotics** | **1988/male** |
| Negative | 0 | 0.6 | 8 | Control post pneumonia | 1961/male |
| Negative | 1 | 3.0 | 38 | Myelodysplasia, Stam cells transplantation, fever, intravenous antibiotics, no infiltration on chest x-ray | 1974/male |
| Negative (antibiotics) | **2** | **3.5** | **43** | **Pneumonia, ongoing intravenous antibiotic** | **1973/male** |
| Negative | **1** | **3.4** | **6** | **Multiple sclerosis, Rituximab, Pneumonia, intravenous antibiotics** | **1981/female** |
| Negative | **2** | **3.46** | **51** | **Aspiration pneumonia, intravenous antibiotics** | **1972/male** |
| *Pseudomonas aeruginosa* | **2** | **3.4** | **44** | **Bronchiectasis, exacerbation, intravenous antibiotics** | **1984/male** |
| Negative | 0 | 2.45 | 35 | Lymphoma, control post pneumonia | 1968/male |
| Negative | 0 | 3.25 | 17 | Lung cancer recently discovered | 1970/male |
| *Negative* | **3** | **3.7** | **116** | **Immunosuppression, bone morrow transplantation, GVH, SIRS, pneumonia, Intensive care unit** | **1983/male** |
| *Streptococcus pneumoniae* | **1** | **3.42** | **58** | **Pneumonia, ongoing intravenous antibiotic therapy** | **1961/female** |
| Negative | 0 | 0.5 | 23 | Control post pneumonia | 1961/male |
| *Pseudomonas aeruginosa x 2, ESBL* | **3** | **3.4** | **127** | **Epilepsy, Cystic fibrosis, pneumonia, deceased** | **1977/female** |
| *Pseudomonas aeruginosa*  *Staphylococcus aureus*  *Candida albicans* | **2** | **3.4** | **59** | **Bronchiectasis, recurrent infection, fever, new infiltration on chest x-ray** | **1984/male** |
| Negative | 1 | 3.4 | 21 | Control post pneumonia | 1973/female |
| Negative | 1 | 3.4 | 37 | Sarcoidosis, green coloured expectoration, judged as no infection | 1974/male |
| *Pseudomonas aeruginosa* | **3** | **3.5** | **123** | **COPD exacerbation, Therapeutic failure broad intravenous antibiotics** | **1961/female** |

**Supplementary Table:** The included cases in the study both infection (bold) and controls. The patients are divided in 2 groups based on the judgement of the physician in charge, documented in the patient reports.
