# Supplementary figures and images for: One-minute through test to distinguish lower respiratory infection by analysis of sputum; exploring the mechanisms
Source: BMC Res Notes. 2018 Sep 12;11:664. doi: 10.1186/s13104-018-3771-1 (PMC6134600; doi:10.1186/s13104-018-3771-1)

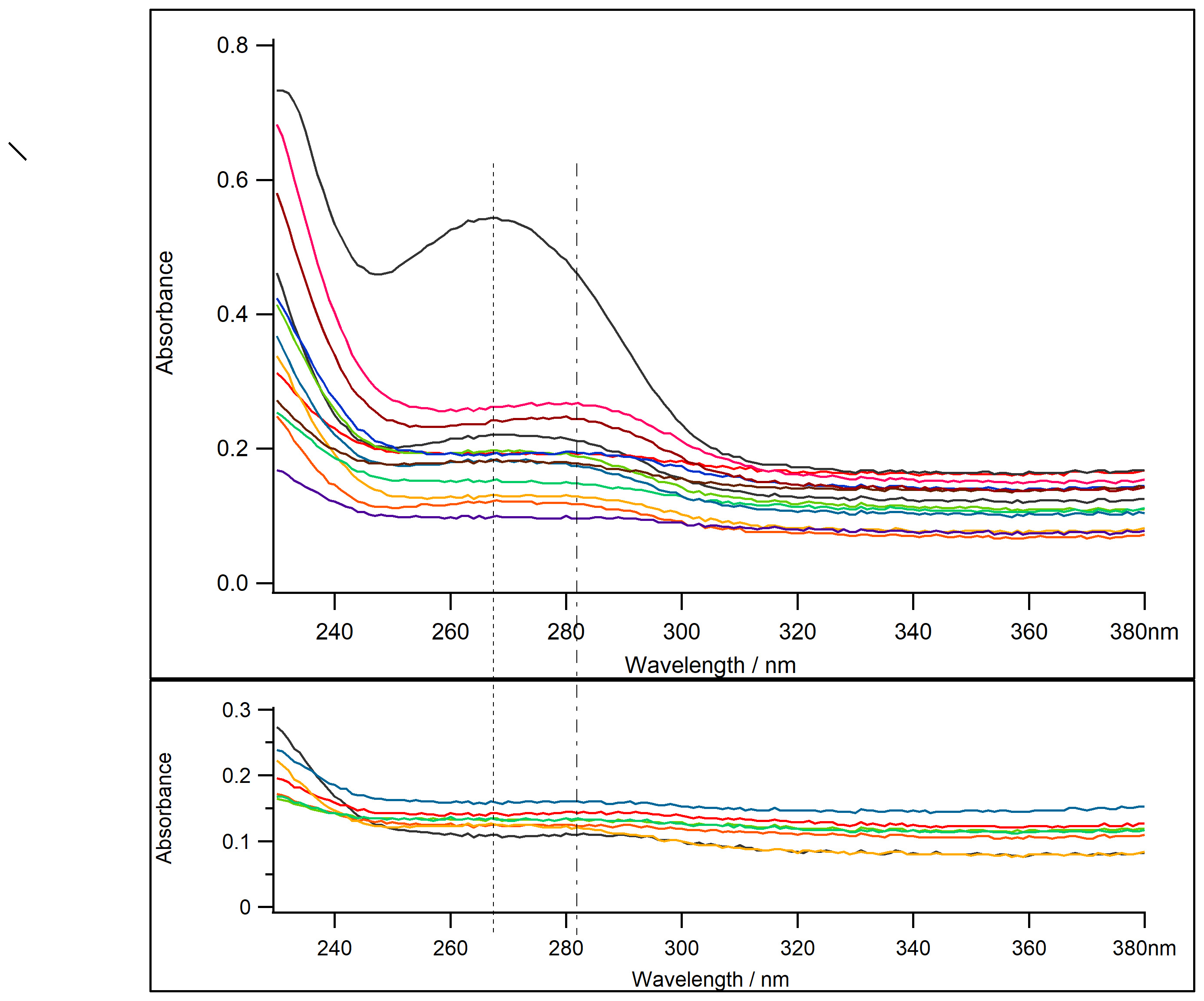

Supplement: Supplementary file 2 — Additional file 2: Figure S1. UV-vis spectrophotometry of sputum samples from patients with respiratory infection (upper image) shows an increased absorbance at 265 nm (chromatin) compared to controls (lower image). [file 13104_2018_3771_MOESM2_ESM.jpg]

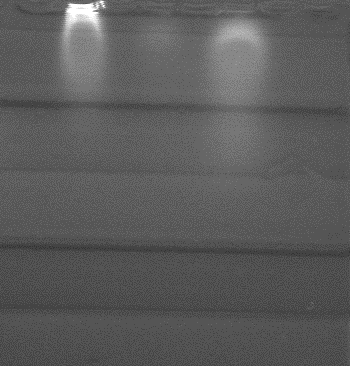

Supplement: Supplementary file 3 — Additional file 3: Figure S2. Agaros gel electrophoresis of samples. The gel was dyed with 1:5000 CYBR green dye for both single and double-strand DNA. The second lane on left is the renentate from the pus from tooth abscess that was centrifuged 60,000 rpm in 60 min in ultra-filtration 30 kDa filters (index test highly positive), the fourth lane on left is 0.5 ml air-dried sputum sample reconstituted in 100 µl MQ (index test positive). The sixth lane on left is the renentate from the sputum sample that was centrifuged 60,000 rpm in 60 min in ultra-filtration 30 kDa filters (index test highly positive). The first lane on the right is the filtrate from same sputum sample (index test negative). The experiment was repeated six times. [file 13104_2018_3771_MOESM3_ESM.tif]
